# The ability of primary healthcare clinics to provide quality diabetes care: An audit

**DOI:** 10.4102/phcfm.v11i1.2094

**Published:** 2019-10-17

**Authors:** Elizabeth M. Webb, Paul Rheeder, Jacqueline E. Wolvaardt

**Affiliations:** 1 School of Health Systems and Public Health, University of Pretoria, Pretoria, South Africa; 2 Department of Internal Medicine, School of Medicine, University of Pretoria, Pretoria, South Africa

**Keywords:** diabetes, primary care, quality, audit, clinic

## Abstract

**Background:**

In South Africa, much of diabetes care takes place at primary healthcare (PHC) facilities where screening for diabetic complications is often low. Clinics require access to equipment, resources and a functional health system to do effective screening, but what is unknown is whether these components are in place.

**Aim:**

The aim of this study was to assess the capacity of primary care clinics in one district to provide quality diabetes care.

**Setting:**

This study was conducted at the Tshwane district in South Africa.

**Methods:**

An audit was done in 12 PHC clinics. A self-developed audit tool based on national and clinical guidelines was developed and completed using observation and interviewing the clinic manager and pharmacist or pharmacy assistant.

**Results:**

Scales, height rods, glucometers and blood pressure machines were available. Monofilaments were unknown and calibration of equipment was rare. The Essential Drug List was the only guideline consistently available. All sites reported consistent access to medication, glucose strips and urine dipsticks. All sites made use of the chronic disease register, and only 25% used an appointment system. No diabetes-specific structured care form was in use. All facilities had registered and enrolled nurses and access to doctors. Availability of educational material was generally poor.

**Conclusion:**

The capacity to deliver quality care is compromised by the poor availability of guidelines, educational material and the absence of monofilaments. These are modifiable risk factors that could be resolved by the clinic managers and staff development educators. However, patient records and health information systems need attention at policy level.

## Introduction

Africa’s population is predicted to reach 2.4 billion by 2050 and coupled with a predicted increase in life expectancy, the prevalence of type 2 diabetes will also rise.^1^ The magnitude of the rise in prevalence is estimated to be a 109% increase in the period 2013–2035 and Africa will experience the largest increase.^2^ The control and management of diabetes is key to prevent complications and the consequent burden on the health system. Complications such as diabetic retinopathy and nephropathy are the leading causes of preventable blindness and end-stage kidney failure in the United States.^3^ In addition, more than 60% of lower limb amputations occur in people with diabetes.^3^ On the contrary, good control of glucose and blood pressure can result in significant reductions of these complications.^4^ A health system that is designed for acute care needs to be adapted for challenges linked to chronic care to meet the ever-increasing demand for chronic disease care. Too often chronic care is characterised by uninformed, passive patients interacting with an unprepared health team, short health visits and little planning to ensure that patients’ needs are addressed.^5^ The chronic care model (CCM) is a useful tool to ensure that all aspects of diabetes care are addressed and patient participation is achieved.^5^ The CCM addresses six essential elements: (1) community resources and policies, (2) healthcare organisation, (3) self-management support, (4) delivery system design, (5) decision support and (6) clinical information systems. For improved chronic care, linkages between the health system and community activities such as exercise programmes, education classes or home-care organisations are important.^5^ The health system structure, goals and values of healthcare providers form the foundation for chronic disease care. Management of the chronic conditions can be taught, and many aspects of the disease can be self-managed, such as diet, exercise, self-measurement, medication use and adherence. Care should be clearly divided across the health system, for example, the responsibilities of the doctor and other health team members with regard to supporting self-management, arranging routine tasks such as laboratory tests, screening examinations for eyes and feet and ensuring follow-up. Planned visits are important in chronic disease care.^6^ Evidence-based clinical guidelines provide healthcare workers with standards of care that need to be integrated into daily practice with a specialist ideally only a phone call away. Computerised registries, as part of clinical information systems, have three important functions: (1) planning patient care (such as ordering blood tests) and planning for the entire patient population, (2) serving as a reminder system for primary healthcare (PHC) teams to comply with practice guidelines and (3) providing feedback to the healthcare professional regarding the individual patient’s care needs. Models such as the CCM are dependent on functional health systems; however, health systems in Africa, and South Africa (SA) in particular, are under pressure because of a substantial concurrent burden of communicable diseases.^7,8^

## Organisation of care in South Africa

South Africa has a population of 54 million (2014), with approximately two-thirds of the population living in urban areas.^9^ The South African health services are jointly managed by the three levels of government, namely, national, provincial and local government. The National Department of Health (NDoH) oversees the delivery of healthcare and sets policy guidelines for service delivery, while PHC services are rendered by both provincial (clinics and community health centres [CHCs]) and local government (clinics). The South African health system consists of both a public and a private sector component. More than 80% of South Africans make use of the public sector. The cornerstone of healthcare delivery in the public sector is through PHC clinics, managed either by local or by provincial health authorities. Services at the PHC clinics are nurse-driven with doctors doing sessional work (usually 4–8 h per week), while CHCs, managed by provincial health authorities, have full-time doctors. Primary healthcare clinics predominately use paper-based patient records and health information systems. Primary healthcare is available at no cost to users and is guided by the use of national standard treatment guidelines and the South African Essential Drug List (EDL).^10^

## Diabetes care in Tshwane district

In 2013, the estimated prevalence of diabetes in SA was 9.3%.^11^ The diabetes epidemic is highest in the Indian population (9% – 11%), followed by 8% – 10% in the mixed race population, 5% – 8% in the black population and 4% in the white population.^9,12^ The prevalence of diabetes in Tshwane district is not known; however, high prevalence rates of poor glycaemic control, blood pressure control and diabetic complications have been reported in this district.^13^ According to the treatment guidelines, diabetic patients should be seen by a health professional (either nurse or doctor) at least 4 times a year. In practice, most diabetic patients attend PHC clinics monthly to have their random glucose, blood pressure and weight checked and to collect their medication. A cluster-randomised trial showed that metabolic and lipid control of patients who attend PHC clinics in Tshwane district was sub-optimal and screening for complications was very low.^13,14,15^ This finding suggests that there are problems with adherence to treatment guidelines in PHC clinics which, in turn, result in poor quality health outcomes.

## Quality of care

Quality of care can be defined as ‘the best health outcomes that are possible, given available resources, and that are consistent with patient values, and preferences’ (p. 146).^16^ Quality of care is multidimensional and has nine dimensions.^17^ These are technical competence, access to service, effectiveness of care, interpersonal relations, efficiency of service delivery, continuity of services, safety, physical infrastructure and choice. Patients, healthcare providers and policymakers may place greater value on different dimensions of quality. For example, healthcare professionals might value technical competence, effectiveness and continuity of care than on other aspects of quality care. In practice, this would translate into ensuring the correct equipment and supplies, an adequate skills mix of health professionals and an efficient referral network. Policymakers, on the contrary, might prioritise efficiency, effectiveness and safety, and would therefore focus on developing policies, treatment algorithms and setting standards. In SA, diabetes primary care is guided by the NDoH’s policy document on diabetes care and management (summarised in Table 1) and the EDL.^10,18^ The Society for Endocrinology, Metabolism and Diabetes, South Africa (SEMDSA) issues additional guidelines for clinical care, but these are optional and are intended to inform care, enhance prevention efforts and reduce complications.^19^

**TABLE 1:** Summary of diabetes treatment policy.

Test	Recommended frequency of tests			
	Each visit	Every 3 months	Every 6 months	Every 12 months
Signs and symptoms	√			
Weight	√			
Blood pressure	√			
Feet examined	√			
Urine glucose/ketones	√			
Blood glucose	√			
HbA1c		√ or	√	
Proteinuria/MACR				√
Urea and creatinine				√
Eyes				√
Nerves/pedal pulses (feet)				√
Lipids				√
ECG				√ (if possible)
MACR				√

*Source*: South African National Department of Health. Diabetes policy guidelines; September 2005

√ , recommended test; MACR, microalbumin–creatinine ratio; ECG, electrocardiogram.

It is essential that PHC clinics meet the national treatment guidelines and standards to ensure quality of care. Standards of care can be divided into structure, process and outcomes.^20^ The evaluation of ‘structural’ standards includes aspects such as physical infrastructure, equipment, staff and medication (in other words material things). All healthcare activities during a patient’s progress through the facility are regarded as ‘process’ standards, while ‘outcomes’ standards evaluate the end results of the care received, successful treatment given, level of control achieved and complications avoided. Indicators can be developed for specific conditions such as diabetes. One example is Diabetes UK that has formulated process and outcome indicators to monitor the quality of diabetes care.^21^ Unfortunately, the South African policy guidelines do not include such a set of indicators. The PHC clinics in the Tshwane study did not achieve optimal metabolic and lipid control and had low screening for complications.^13,14,15^ Achieving the standards for quality diabetes care is only possible if the correct equipment, staff, medication, clinical records and so on are available.

The aim of this study was to assess the capacity of participating clinics to provide quality diabetes care, as defined by the clinical guidelines outlined in the national diabetes policy, the EDL and SEMDSA guidelines.

## Methods

### Study design

A facility audit was conducted in 2013 as part of a cluster-randomised trial that was conducted among the PHC clinics of Tshwane district.

### Setting

Tshwane district has a population of 2.9 million and is in the highly urbanised province of Gauteng.^22^ Within the Tshwane district, healthcare in the public sector is delivered by 86 health facilities: 64 PHC clinics, 8 community health centres, 3 satellite clinics, 5 district hospitals, 1 regional hospital, 1 tertiary hospital and 4 specialist hospitals.

### Study population and sample strategy

A list was compiled of the 64 PHC clinics and 8 CHCs in the Tshwane district from the two management levels for clinic-based healthcare within the public sector. These included clinics managed by local government as well as clinics and CHCs managed by the provincial government. Four clinics were randomly selected from each of the two levels of management and thus three clinic types, totalling 12 clinics.

### Data collection

An audit tool was self-developed based on the national diabetes policy, SEMDSA and the EDL guidelines. The tool was used to assess whether the PHC clinics meet the structural standard needed to achieve the required technical competence. Data were collected by conducting face-to-face individual interviews with either the clinic manager, the professional nurse dedicated to chronic disease management and the clinic’s pharmacist or pharmacy assistant. The tool focussed on eight domains: (1) equipment, (2) guidelines and health promotion material, (3) medication for diabetes and co-morbidities, (4) record-keeping, (5) staff establishment, (6) diabetes education material, (7) support structures and (8) referral network.

### Data analysis

Analyses were done as proportions and counts because of the small sample size of 12 clinics. Data from the clinic audit tool were captured in Epidata (version 3.1, 2008) and analysed using STATA Corp (version 13.1, 2013).

### Ethical consideration

Informed consent was obtained from healthcare providers working in the clinics where the study was conducted. The study was approved by the University of Pretoria’s Faculty of Health Sciences Research Ethics Committee (Protocol # 61B/2010), by the Tshwane Metropolitan Council and by the Tshwane Metsweding Region Research Ethics Committee (Project # TMREC 2010/19).

## Results

### Equipment

All 12 clinics reported that they have scales and height rods. Ten of the 12 clinics (83%) had a combined machine. Almost all of the clinics (83%, *n* = 10) had a tape measure for waist circumference measurement and all clinics had glucometers. All clinics had blood pressure machines and obese cuffs were available in 11 clinics (92%). Seven clinics (58.3%) had electrocardiogram (ECG) machines, and body mass index (BMI) wheels were available in less than half the clinics (41.7%, *n* = 5). Nine clinics (75%) had an ophthalmoscope and eight (66.7%) had Snellen charts. Two-thirds of the clinics (66.7%, *n* = 8) had tuning forks and almost all the clinics (91.7%, *n* = 11) had patella hammers. No clinics had monofilaments available. Only one clinic reported that they have their ECG machine calibrated yearly.

### Guidelines

The SEMDSA guidelines were not known, nor available, in any clinic, but the EDL was available in all facilities. The Department of Health guidelines on primary care for diabetes were available in seven clinics (58%). Other guidelines found in three of the clinics (25%) included ‘Foot Care Guidelines for People with Diabetes’ from the Provincial Department of Health, and a self-compiled dietary guidelines for diabetic patients in one clinic (8%).

### Medication

All clinics reported that they had access to all the medication as prescribed by the national treatment algorithms. Two clinics (16.7%) reported a stock-out of simvastatin for a duration of 2 months in the 12 months prior to the study. All clinics reported that urine dipsticks and glucose test strips were always available, but that neither blood glucose strips nor urine glucose strips are available on the EDL for patients’ use at home.

### Record-keeping

All clinics had a chronic disease register in use, but no diabetes-specific structured care form. Only two clinics (17%) had a special sticker on the front of a patient’s file indicating a diabetes diagnosis. In all clinics, the patient-retained card indicates a return date, but this is not captured in the clinic in an appointment register and the responsibility remains with the patient to come back for the follow-up visit. Only one quarter (25%, *n* = 4) of the clinics made use of an appointment system.

### Staff

All clinics had both professional and enrolled nurses and all had a consulting doctor for four or more hours per week for all complicated cases (not necessarily diabetes-related) or emergencies. Seven clinics (58%) had a full-time doctor. Eight clinics (67%) had a health promotion officer, seven of whom were employed full-time at the clinic. Only one clinic (8%) had a qualified diabetes educator. Two clinics (17%) had a full-time dietician, while patients in five clinics (42%) only had intermittent access to a dietician. In four of these clinics, the dietician visited the clinic once a month, while in the fifth clinic the dietician was only available if needed. No clinics had a nutrition advisor. Seven clinics (58%) reported that their patients had access to a podiatrist, either through a monthly routine visit or else via the referral system. Two clinics (17%) had full-time podiatrists on their staff establishment.

Other staff members listed as part of the healthcare team, including pharmacy assistants, health promoters, lay counsellors and a social worker. One clinic (8%) had a trained ophthalmic nurse. Two clinics (17%) were serviced by a private optometrist with a mobile service offering visual acuity screening.

### Diabetes education

All clinics reported that individual education of diabetes patients is done at every visit, with every patient by the consulting nurse or doctor. Eight clinics (67%) also run group sessions for diabetes education. The majority (50%) of these sessions are held monthly (compared to 33% weekly and 17% daily) and are facilitated by health promoters. The method of health education activities included talks (67%, *n* = 8), pamphlets (58%, *n* = 7), posters (50%, *n* = 6) and videos or DVDs (33%, *n* = 4). Five clinics (42%) had a diabetes poster somewhere in the clinic, and four clinics (33%) reported that they have books or booklets on diabetes available for patients.

### Support structures

Four of the 12 clinics had a diabetes support group and one planned a support group. Other support systems listed were a weekly church gathering for people with chronic diseases, a vegetable garden managed by patients and a home visit nurse.

### Referral network

All clinics reported that they have a structured referral network and all respondents could name their referral hospital. Ten clinics (83%) reported that referring a patient is easy, but that there is a lack of feedback to the clinic. Two clinics (17%) listed an absence of transport as a barrier to refer patients. Only two clinics (17%) reported that they do not have access to either a telephone or a fax machine.

## Discussion

This study gives an initial insight into the capacity of the PHC clinics – an often overlooked aspect of providing quality diabetes care – to provide diabetes care.

It is clear that the PHC clinics had much of the equipment needed to achieve the technical performance required. Quality screening for complications – as measured by *any* one element (e.g. either an ophthalmoscope or a Snellen chart) being available in the clinic to evaluate care for heart health, eyes, feet or kidneys – would have only been possible in half of the facilities in this study. These items are essential for quality diabetes care that meets the national treatment guidelines. While the BMI can be calculated in other ways, nothing can replace either the tuning forks used to test vibration sense or the monofilaments to test for neuropathy. The absence of the monofilaments is a particular concern as the NDoH standard for foot care is a yearly foot examination.

The official NDoH guidelines were absent in more than a third of the clinics. This absence is a concern as it is this document that sets the standards to ensure quality diabetes care. None of the clinics knew about the SEMDSA guidelines, which is a possible indication of the low profile of diabetes care in PHC settings. One positive finding was that all the clinics had the EDL available.

All clinics had all the medication required by the treatment guidelines with only a few reported stock-outs. Although urine dipsticks and glucose test strips were always available for staff, the EDL does not make these available for patients’ home care. Patients on insulin are the patients with the worst diabetes-related outcomes in PHC settings.^14^ Testing for ketones in the urine to prevent a diabetic ketoacidosis incident or testing the random glucose to adjust the insulin dose are tools for self-management and a key element of the CCM. The absence of urine dipsticks and glucose test strips for home care negatively affects patients’ ability to take responsibility for his or her own disease management.

Although all clinics made use of the chronic disease register, this register does not allow for the capture of clinical information such as screening for diabetic complications or other risk factors such as smoking, diet and exercise. The content fields of the register are determined by the NDoH and its primary purpose is to collect data regarding a count across all the chronic diseases. Because of a lack of denominator data it is not possible to calculate prevalence of the different diseases. The current register does not meet the requirements of a clinical information system as outlined by the CCM because this model describes the registry as a tool to plan individual patient care and conduct population-based care.^5^ All three roles of the clinical information system described by the CCM are dependent on computerised information, which is not yet a reality in the South African environment. Besides the registry as a planning tool, the reminder system as part of structured care for healthcare professionals regarding routine tests, for example, the annual eye test and the necessary feedback for clinical management of the patient by the treating nurse or doctor, was also absent.^6^ Two clinics tried to create a rudimentary clinical information system by adding a sticker to the patients file. No diabetes-specific structured care form was found to be in use in any of the clinics.^6^

The delivery system design of the CCM suggests that practice teams with a clear division of labour and the inclusion of non-physician personnel are needed.^6^ In this study, all clinics had multi-disciplinary professional teams that included a wide range of non-physician personnel. Eye care services were the most sparsely provided.

To achieve self-management support as outlined in the CCM, patients need information to enable them to become their own primary caregiver.^6^ All the clinics reported that education of diabetes patients is done individually at every consultation and the majority also had monthly group sessions. However, the availability of patient education material was generally poor.

Continuity of services was ensured via the structured referral network, but the relative ease of referral was offset by the complaint of the lack of feedback after the referral. This lack of feedback to primary healthcare facilities is common.^23^

The findings of this study partially explain the lack of complication screening reported by Webb et al.^13,14,15^

## Strengths and limitations

The audit provided information on PHC clinics’ ability to provide quality diabetes care, an aspect that is mostly overshadowed by the higher profile conditions of HIV and TB. The small size (12 clinics) of the sample limits the generalisability of the results. We recommend studies, using methodologies such as action research, to explore the use of quality improvement strategies to address these service delivery gaps.

## Conclusion

Within health systems some factors are within the scope of practice of clinic managers, and therefore modifiable, while others are not. In this study, some of the barriers to effective diabetes care such as paper-based patient records and the limitations of the chronic disease register are beyond the reach of clinic managers. From a health system perspective, the findings regarding the referral system and the good supply of essential medicines are positive. However, the referral system is flawed, as the absence of regular feedback to the PHC clinics is both a lost opportunity to learn and an obstacle to ensure continuity of care. In this study, the provision of a multi-disciplinary team was uneven and could be as a result of the different kinds of settings (PHC clinic vs. community health centre) that have different staff establishments.

On the contrary, the relatively poor availability of the treatment guidelines, posters, patient education material, support groups, tuning forks, BMI wheels and the complete absence of monofilaments is within the control of a PHC manager as these resources are available to them. Ensuring the correct material, equipment and supplies is a key competence of any PHC manager and one that directly contributes to ensuring quality of care.
